# Eurasian golden jackal as host of canine vector-borne protists

**DOI:** 10.1186/s13071-017-2110-z

**Published:** 2017-04-14

**Authors:** Barbora Mitková, Kristýna Hrazdilová, Gianluca D’Amico, Georg Gerhard Duscher, Franz Suchentrunk, Pavel Forejtek, Călin Mircea Gherman, Ioana Adriana Matei, Angela Monica Ionică, Aikaterini Alexandra Daskalaki, Andrei Daniel Mihalca, Jan Votýpka, Pavel Hulva, David Modrý

**Affiliations:** 1 Department of Pathology and Parasitology, University of Veterinary and Pharmaceutical Sciences, Palackého tr. 1946/1, 612 42 Brno, Czech Republic; 2 CEITEC-VFU, University of Veterinary and Pharmaceutical Sciences, Palackého tr. 1946/1, 612 42 Brno, Czech Republic; 3 Department of Virology, Veterinary Research Institute, Hudcova 296/70, 621 00 Brno, Czech Republic; 4 Department of Parasitology and Parasitic Diseases, Faculty of Veterinary Medicine, University of Agricultural Sciences and Veterinary Medicine Cluj-Napoca, Calea Mănăștur 3-5, 400372 Cluj-Napoca, Romania; 5 Institute of Parasitology, Department of Pathobiology, University of Veterinary Medicine Vienna, Veterinaerplatz 1, 1210 Vienna, Austria; 6 Research Institute of Wildlife Ecology, Department of Integrative Biology and Evolution, University of Veterinary Medicine Vienna, Savoyenstraße 1, 1160 Vienna, Austria; 7 Animal Protection, Welfare and Behaviour, University of Veterinary and Pharmaceutical Sciences, Palackého tr. 1946/1, 612 42 Brno, Czech Republic; 8 Central European Institute of Game Ecology, Šumavská 416/15, 602 00 Brno, Czech Republic; 9 Department of Parasitology, Charles University in Prague, Viničná 7, 128 44 Prague, Czech Republic; 10 Biology Centre, Institute of Parasitology, Czech Academy of Sciences, Branišovská 31, 37005 České Budějovice, Czech Republic; 11 Department of Zoology, Charles University in Prague, Viničná 7, 128 44 Prague, Czech Republic; 12 Department of Biology and Ecology, University of Ostrava, Dvořákova 7, 701 03 Ostrava, Czech Republic

**Keywords:** Eurasian golden jackal, *Babesia*, *Hepatozoon*, “Theileria annae”, *Leishmania*

## Abstract

**Background:**

Jackals are medium-sized canids from the wolf-like clade, exhibiting a unique combination of ancestral morphotypes, broad trophic niches, and close phylogenetic relationships with the wolf and dog. Thus, they represent a potential host of several pathogens with diverse transmission routes. Recently, populations of the Eurasian golden jackal *Canis aureus* have expanded into the Western Palaearctic, including most of Europe. The aim of our study was to examine Eurasian golden jackals from Romania, Czech Republic and Austria for a wide spectrum of vector-borne protists and to evaluate the role of this species as a reservoir of disease for domestic dogs and/or humans.

**Results:**

Diagnostic polymerase chain reaction (PCR) DNA amplifications revealed 70% of jackals to be positive for *Hepatozoon*, 12.5% positive for piroplasms, and one individual positive for *Leishmania infantum*. Phylogenetic analyses of partial *18S rDNA* sequences invariably placed sequenced isolates of *Hepatozoon* into the *H. canis* clade. For piroplasms, both the *18S* and *cox*1 sequences obtained confirmed the presence of *Babesia canis* and “Theileria annae*”* in 5 and 2 individuals, respectively, providing the first records of these two piroplasmids in Eurasian golden jackals. A single animal from Dolj County (Romania) was PCR-positive for *L. infantum,* as confirmed also by sequencing of *ITS1-5.8S*.

**Conclusions:**

Apparently, expanding populations of jackals can play a significant role in spreading and maintaining new *Babesia canis* foci in Central Europe. The role of jackals in the epidemiology of “Theileria annae” and *H. canis* is probably similar to that of red foxes and should be taken into account in further research on these parasites. Also the presence of *L. infantum* deserves attention. Our study confirms that once established, the populations of Eurasian golden jackals constitute natural reservoirs for many canine vector-borne diseases, analogous to the role of the coyotes in North America.

## Background

Wildlife reservoirs for diseases of companion animals and humans merit serious attention (e.g., [1–4]), due to recent dynamics in their populations and distribution ranges [5] and the resulting changes in disease epidemiology. High population densities and ubiquitous presence make red foxes (*Vulpes vulpes*) the most commonly discussed wild carnivore in Europe. However, the current dramatic expansion of Eurasian golden jackal [6–8] is likely to change the situation. In contrast to the red fox, the Eurasian golden jackal is closely related to and may hybridize with the domestic dog [9]. A preference for lowland habitats, ability to establish vital populations near human settlements and close phylogenetic relationships with dogs increase the probability of interactions between these two canid species. Considering these facts together, Eurasian golden jackals might become the most significant reservoir of diseases of zoonotic and veterinary importance among the European canids. The Eurasian golden jackal has been reported as a host of pathogens of zoonotic and/or veterinary importance, including a range of vector-borne pathogens such as *Ehrlichia canis*, *Anaplasma phagocytophilum*, the spotted fever group rickettsiae [10], *Dirofilaria* spp. [11], and recently also *Thelazia callipaeda* [12]. Among tick-borne protists, *Hepatozoon canis* is the only pathogen reported in golden jackals in Europe [13, 14]. There is a near absence of published data regarding piroplasms in Eurasian golden jackals. Several studies have reported the presence of *Leishmania infantum* in Eurasian golden jackals [15, 16] and suggested their importance as a potential reservoir of zoonotic *L. infantum*.

The aim of our study was to examine tissues of Eurasian golden jackals from Romania, Czech Republic and Austria for a spectrum of vector-borne protists and to evaluate the role of this species as a potential reservoir of diseases for domestic dogs and/or humans. Using phylogenetic analyses of nuclear and mitochondrial markers, we provide more detailed insight into parasites’ intraspecific variability, ruling out possible existence of jackal-specific lineages of the parasites investigated.

## Methods

Fifty-four Eurasian golden jackals were legally hunted between October 2013 and May 2015 in 11 counties of Romania with established jackal populations. To sample animals from the edge of distribution range, we also included a single available animal from the Czech Republic, shot in the Moravia-Silesia Region near Nový Jičín (July 2014), and a single Austrian road-killed Eurasian golden jackal from Wiener Neudorf in Lower Austria (January 2012). All carcasses were frozen and transported to the laboratory for necropsy, wherein blood, blood clot and spleen samples were collected and frozen at -20 °C. Bone marrow used for *Leishmania* detection was collected and frozen from a subset of 36 Romanian golden jackals only.

### DNA isolation and polymerase chain reaction

DNA from Romanian and Czech golden jackal blood samples was isolated using the Genomic DNA Mini Kit (Geneaid Biotech, Taipei city, Taiwan) from 200 μl of blood or blood clot equivalent in size. In one Romanian animal in which blood was not available, 20 mg of spleen tissue were used for DNA isolation. In a subset of 36 golden jackals, DNA was isolated from 20 mg of bone marrow using a High Pure PCR Template Preparation Kit (Roche Life Science, Mennheim, Germany). The processing of the Austrian sample is described elsewhere [13].

#### Hepatozoon and Piroplasmida PCR

All 56 samples were screened using two different diagnostic PCRs amplifying fragments of *Hepatozoon* spp. (~670 bp) or piroplasms (~560 bp) small ribosomal subunit (*18S rDNA*) to identify the positive ones.

Genus specific primers were used for *Hepatozoon* spp. detection [17]. For positive samples, further PCR was performed to obtain ~1700 bp fragments of *18S rDNA* [18].

For piroplasms detection nested PCR was used [19]. For positive samples, modified nested PCR amplifying ~1700 bp fragments of *18S rDNA* using the combination of BT1 F, BT outer R primers in first run and BT Inner R [20] and Piro0F2 [21] primers in second run was used. To confirm the determination of piroplasmids and to obtain further phylogenetic characterization, two modified nested PCR protocols amplifying fragments of cytochrome *c* oxidase subunit 1 (*cox*1*)* gene were performed [21, 22]. Table 1 provides details on the primers used, PCR conditions and obtained fragments for all PCRs.

**Table 1:** Details for PCR protocols used

Parasite	Gene	Fragment length (bp)	Primer	Primer sequence (5’-3’)	PCR conditions, master mix used	Source
*Hepatozoon* spp.	18S	~670	Hep F	ATACATGAGCAAAATCTCAAC	94 °C for 3 min, 35× (94 °C for 1 min, 61 °C for 1 min, 72 °C for 2 min), 72 °C for 7 min	[17]
			Hep R	CTTATTATTCCATGCTGCAG	PPP Master Mix (TopBio s.r.o., Czech Republic)	
	18S	~1765	HAM 1 F	GCCAGTAGTCATATGCTTGTC	95 °C for 5 min, 35× (95 °C for 20 s, 56 °C for 1 min, 72 °C for 1 min), 72 °C for 5 min	[18]
			HPF 2R	GACTTCTCCTTCGTCTAAG	PCRBIO Taq DNA Polymerase (PCR Biosystems Ltd, UK)	
Piroplasmida	18S	~685	BTH 1 F	CCTGAGAAACGGCTACCA CATCT	95 °C for 10 min, 40× (95 °C for 30 s, 60 °C for 1 min, 72 °C for 1 min), 72 °C for 10 min	[19]
			BTH 1R	TTGCGACCATACTCCCCCCA		
		~560	GF2	GTCTTGTAATTGGAATGA TGG	95 °C for 10 min, 40× (95 °C for 30 s, 62 °C for 1 min, 72 °C for 1 min), 72 °C for 10 min	
			GR2	CCAAAGACTTTGATTTCTCTC	PPP Master Mix (TopBio s.r.o., Czech Republic)	
	18S	~1730	BT1 F	GGTTGATCCTGCCAGTAGT	94 °C for 30 s, 20× (94 °C for 30 s, 65-55 °C (-0.5 °C/cycle) for 30 s, 68 °C for 1 min), 20× (94 °C for 30 s, 55 °C for 30 s, 68 °C for 1 min), 68 °C for 5 min	[20]
			BT outer R	GGAAACCTTGTTACGACTTCTC		
		~1670	Piro0F2	GCCAGTAGTCATATGCTTGTCTTA	94 °C for 30 s, 20× (94 °C for 30 s, 65-55 °C (-0.5 °C/cycle) for 30 s, 68 °C for 1 min), 20× (94 °C for 30 s, 55 °C for 30 s, 68 °C for 1 min), 68 °C for 5 min	[21]
			BT Inner R	TTC TCC TTC CTT TAA GTG ATA AG	OneTaq 2x Master Mix with standard buffer (NEB Inc., USA)	[20]
*Babesia* spp.	*cox*1	~1250	Bab_For1	ATWGGATTYTATATGAGTAT	95 °C for 1 min, 35× (95 °C for 15 s, 45 °C for 30 s, 72 °C for 30 s), 72 °C for 10 min	[22]
			Bab_Rev1	ATAATCWGGWATYCTCCTTGG		modified
		~975	Bab_For2	TCTCTWCATGGWTTAATTATGATAT	95 °C for 1 min, 35× (95 °C for 15 s, 45 °C for 30 s, 72 °C for 30 s), 72 °C for 10 min	
			Bab_Rev2	TAGCTCCAATTGAHARWACAAAGTG	PCRBIO Taq DNA Polymerase (PCR Biosystems Ltd, UK)	
*Theileria* spp.	*cox*1	~1020	Cox1F133	GGAGAGCTAGGTAGTAGTGGAGATAGG	95 °C for 1 min, 35× (95 °C for 15 s, 63 °C for 15 s, 72 °C for 30 s), 72 °C for 10 min	[21]
			Cox1R1130	GTGGAAGTGAGCTACCACATACGCTG		
		~985	Cox_cladeI_Fw2	GTAGTGGAGATAGGTTCATAGC	95 °C for 1 min, 35× (95 °C for 15 s, 55 °C for 15 s, 72 °C for 30 s), 72 °C for 10 min	This study
			Cox_cladeI_Rev2	TGTATCGTGTAGTGACACGTC	PCRBIO Taq DNA Polymerase (PCR Biosystems Ltd, UK)	
*Leishmania* spp.	ITS1	~280	ITS-219 F	AGCTGGATCATTTTCCGATG	qPCR: 95 °C for 5 min, 45× (95 °C for 5 s, 57 °C for 15 s, 72 °C for 15 s), melting from 60 to 95 °C at 1 °C/s	[26]
			ITS-219R	ATCGCGACACGTTATGTGAG	iQSYBER Green Supermix (BioRad Inc., USA)	
	ITS1-5.8S	~300	LITSR	CTGGATCATTTTCCGATG	95 °C for 3 min, 40× (95 °C for 20 s, 53 °C for 35 s, 72 °C for 60 s), 72 °C for 5 min	[27]
			L5.8S	ACACTCAGGTCTGTAAAC		
					EmeraldAmp (TaKaRa Clontech, Japan)	

All PCRs were performed using commercial master mix following the manufacturer’s instructions. A total volume of 25 μl was prepared for each reaction containing 12.5 μl of master mix, 10 pmol of each primer, 2 μl of template DNA or 1 μl of PCR product from the first run in the case of nested PCR, plus PCR water. DNA samples from a fox naturally infected by *Hepatozoon* and from a dog naturally infected with *B. gibsoni*, were used as positive controls; in both cases, the infection was confirmed microscopically.

Products from selected PCRs (~1700 bp of *18S rDNA* of *Hepatozoon* PCR and products from second runs of all piroplasmida PCRs) were purified using a Gel/PCR DNA Fragments Extraction Kit (Geneaid Biotech Ltd., Taipei city, Taiwan) and directly sequenced using the amplification primers. For sequencing, a ~1700 bp fragment of *Babesia 18S rDNA*, the additional sequencing primers BTH-1 F, BTH-1R, 600 F, 1200 F [20] were used and one more sequencing primer 18S-R4 (5’-CTT GCG CAT ACT AGG CAT TCC TCG-3’) was designed to cover the whole length of *18S* amplified fragment during the sequencing. Because of low quality of obtained sequences of “T. annae” *cox*1 fragments, PCR products from second run of nested PCR were cloned using a TOPO®-TA Cloning Kit (Thermo Fisher Scientific, Carlsbad, USA). Plasmids from cloning reactions were isolated using a GenElute™ Plasmid Miniprep Kit (Sigma-Aldrich, St. Louis, USA) and sequenced according to the manufacturer’s instructions. All sequencing reactions were done by Macrogen capillary sequencing services (Macrogen Europe, Amserdam, the Netherlands).

#### *Leishmania* PCR

Based on our previous experiences [23, 24] and the extensive review focused on molecular diagnosis of *Leishmania* infections [25] we have decided to use two different PCR protocols (amplifying the same target locus) for the *Leishmania* DNA screening of the subset of 36 samples. Quantitative PCR (iQSYBER Green Supermix, BioRad, Hercules, Foster City, CA, USA) was performed using the primers ITS-219 F and ITS-219R to amplify a ~280 bp product of the internal transcribed spacer (*ITS1*) region of the *Leishmania* rRNA operon [26]. To confirm the results of qPCR obtained, the same 36 DNA samples were also analysed by conventional PCR to amplify a ~300 bp *ITS1-5.8S* fragment using primers LITSR and L5.8S [27]. The DNA of *Leishmania major* was used as a positive control. The conventional PCR products were visualized on 2% agarose gels, purified with a High Pure PCR Product Purification Kit (Roche) and submitted for direct DNA sequencing (ABI 3730 automated sequencer, Applied Biosystems) to the Center for Genomic Technologies at the Faculty of Science, Charles University in Prague.

### Sequence analysis

All obtained sequences were edited and analysed using Geneious® 9.1.2 [28] and compared with those available in the GenBank database by BLASTn analysis (http://blast.ncbi.nlm.nih.gov/Blast.cgi). Alignments of non-coding (*18S rDNA*) sequences were generated using the ClustalW algorithm [29]. For *cox*1 sequences, the nucleotide dataset was translated into amino acids and aligned in BioEdit 7.0.5.3 [30] using the ClustalW algorithm and aligned sequences were translated back to nucleotides. Nucleotide sequences in the edited dataset were then used to infer phylogeny. Evolution models were determined by a likelihood ratio test using R software (R Core Team, 2012). Phylogenetic analyses were performed using the maximum likelihood method in PhyML 3.0 software [31]. Phylogenetic trees were visualized and edited in FigTree v1.4.1 (http://tree.bio.ed.ac.uk/software/figtree/).

## Results

**Fig. 1:**
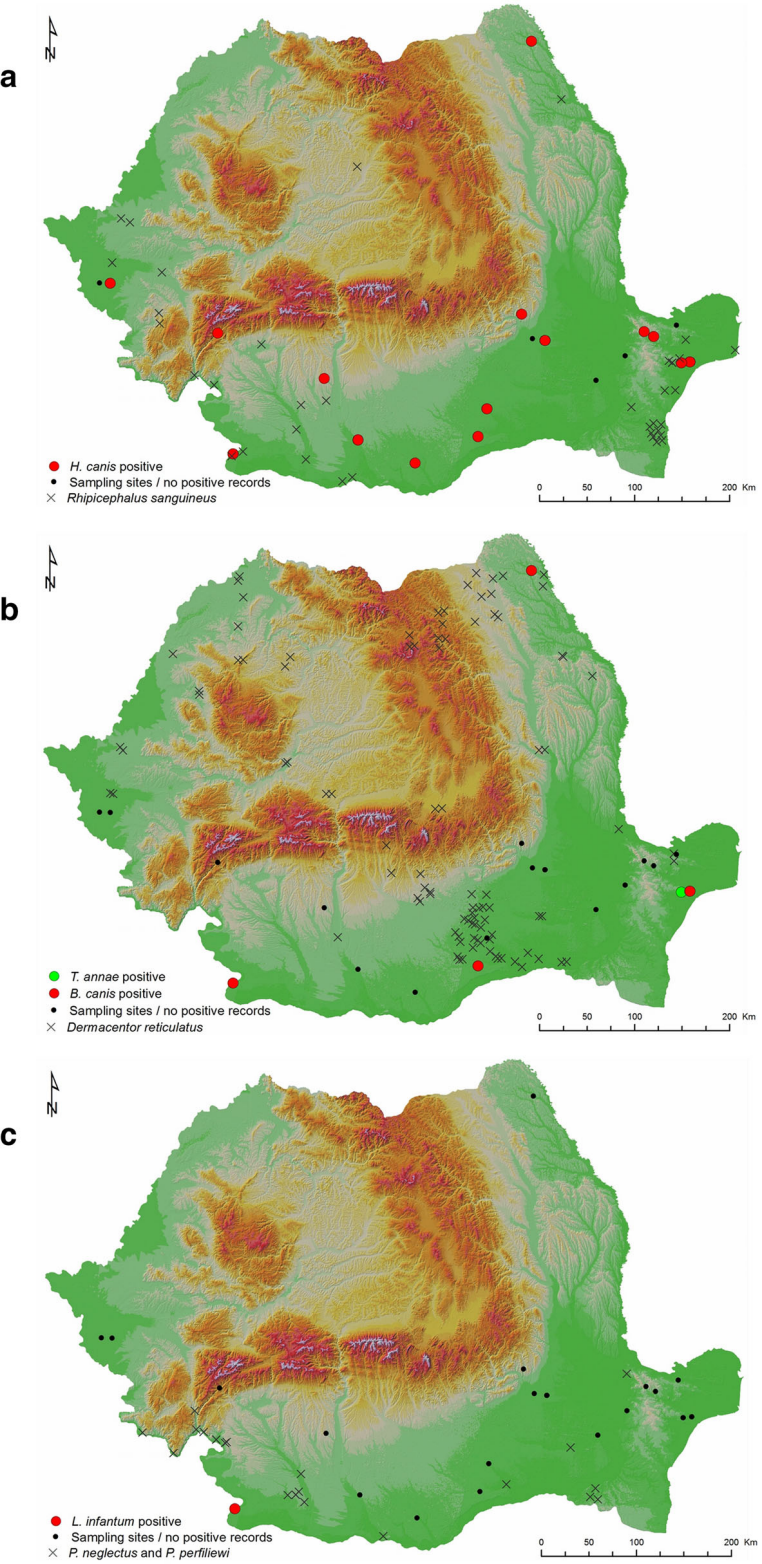
Sampling localities in Romania with positive (*red* and/or *green* dots) or negative (black dot) results. **a** *Hepatozooncanis*; occurrence of *Rhipicephalus sanguineus* (*s.l.*) is marked with an × (Mihalca et al. [39]. **b** Piroplasmida (*Babesia canis*, *red*, “Theileria annae”, *green*); occurrence of *Dermacentor reticulatus* is marked with an × (Mihalca et al. [39]). **c** *Leishmania infantum*; occurrence of *Phlebotomus neglectus* and *Phlebotomus perfiliewi* is marked with an × (Dumitrache et al. [49])

Diagnostic PCR revealed that 39/56 (70%) of the golden jackals were positive for *Hepatozoon*, including the specimens from the Czech Republic and Austria. All seven (12.5%) piroplasm-positive animals originated from Romania and were co-infected with *Hepatozoon.* Furthermore, one sample from Dolj County (Romania) was PCR-positive for *L. infantum* in both protocols used, as confirmed by the sequence of the partial *ITS1-5.8S* fragment. Table 2 and Fig. 1 provide data for the distribution of the positive animals in Romania and Table 3 provides details of the nearest hits in a BLAST search.

**Table 2:** Distribution of positive animals and total number of sampled animals from all localities

	RO											RO	CR	AUS
	BR	BT	BZ	DJ	GR	IF	IL	OT	TL	TM	VL	total	MSK	NÖ
*H.can*is	0/1	0/1	13/18	3/8	0/1	2/2	0/2	2/2	10/16	1/2	1/1	32/54	1/1	1/1
*B. canis*	0/1	0/1	0/18	0/8	0/1	0/2	0/2	0/2	0/16	0/2	0/1	0/54	0/1	0/1
“T. annae”	0/1	0/1	0/18	0/8	0/1	0/2	0/2	0/2	0/16	0/2	0/1	0/54	0/1	0/1
*L. infantum*	0/1	0/1	0/9	1/6	0/1	0/2	0/2	0/2	0/9	0/2	0/1	1/54	0/1	0/1
*B. canis + H. canis*	0/1	1/1	0/18	1/8	1/1	0/2	0/2	0/2	2/16	0/2	0/1	5/54	0/1	0/1
“T. annae” *+ H. canis*	0/1	0/1	0/18	0/8	0/1	0/2	0/2	0/2	2/16	0/2	0/1	2/54	0/1	0/1

*Abbreviations*: *RO* Romania; *BR* Brăila; *BT* Botoşani; *BZ* Buzău; *DJ* Dolj; *GR* Giurgiu; *IF* Ilfov; *IL* Ialomița; *OT* Olt; *TL* Tulcea; *TM* Timiș; *VL* Vâlcea; *CR* Czech Republic; *MSK* Moravskoslezský kraj (Moravian-Silesian Region); *AUS* Austria; *NÖ* Niederösterreich (Lower Austria)

**Table 3:** Sequences obtained in this study and their nearest BLAST hits

	Origin	Gene	Fragment length (bp)	BLAST identity (%), accession no.	Host, origin of GenBank sequence
*Hepatozoon* spp.	RO	18S	~1765	99% *H. canis* AY150067	red fox, Spain
		18S	~1765	99% *H. canis* AY461378	dog, Spain
	CR	18S	~1765	99% *H. canis* AY 461376	pampas fox, Brazil
	AUS	18S	~1765	100% *H. canis* AY150067	red fox, Spain
Piroplasms	RO	18S	~560	99–100% *B. canis* KT008057	dog, Estonia
		18S	~560	99–100% “B. annae” KT580785	red fox, Great Britain
		18S	~1670	99% *B. canis* AY072926	dog, Croatia
		*cox*1	~975	99–100% *B. canis* KC207822	no data
		*cox*1	~985	97% *B.* cf. *microti* KC207827	no data
*Leishmania* spp.	RO	ITS1	~300	100% *L. infantum* AJ000288	no data

*Abbreviations*: *RO* Romania; *CR* Czech Republic; *AUS* Austria

**Fig. 2:**
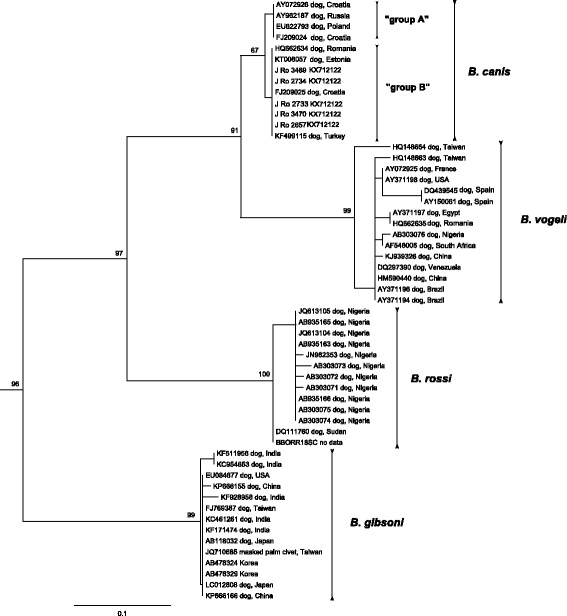
Maximum likelihood tree based on 504 nt-long alignment of 18S rDNA sequences of dogs infected by *Babesia* spp.; sequences of *B. caballi* (AY309955, EU888901, EU642514) used as the outgroup are not shown; bootstrap values from 1000 replicates shown only above 60%

**Fig. 3:**
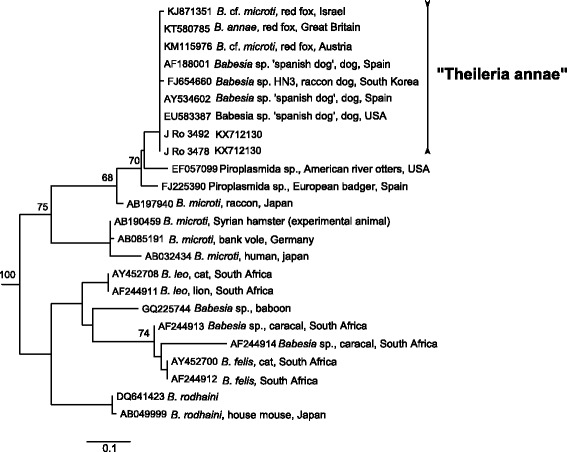
Maximum likelihood tree based on 506 nt-long alignment of 18S rDNA sequences of “clade I” (according to Schnittger et al. [34]); sequences of *B. conradae* (AF158702) and *B. lengau* (GQ411415, AF158700) used as the outgroup are not shown; bootstrap values from 1000 replicates shown only above 60%

The BLAST analysis of partial *18S rDNA* sequences retrieved from piroplasm diagnostic PCR identified 5 positive samples as *Babesia canis* (99–100% identity) and 2 as “Theileria annae” (100% nucleotide identity) (Table 3). Additionally, the ~1700 bp fragments were amplified from two *B. canis*-positive golden jackals, both confirming the *B. canis* genotype B [32, 33] in BLAST. Phylogenetic analysis of 504 bp fragment of *18S rDNA* of *Babesia* (*sensu stricto*) (*s.s*.) [34] species infecting dogs placed all *B. canis* sequences retrieved from golden jackals into “group B” (Fig. 2), together with sequences from domestic dogs. Phylogenetic analysis of 506 bp of *18S rDNA* sequences of piroplasmid clade I according to Schnittger et al. [34] placed both “T. annae” sequences from our samples into a common clade with sequences from foxes and dogs, forming a sister clade to *Babesia microti* sequences, being more distantly related to *B. leo* and *B. rodhaini* (Fig. 3).

**Fig. 4:**
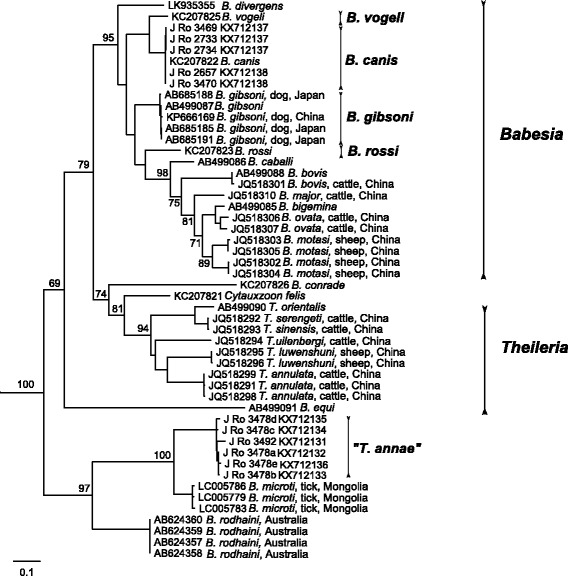
Maximum likelihood tree based on 768 nt-long alignment of *cox*1 sequences of all piroplasms; sequences of *Plasmodium* sp. (AY791691, AB550280, KJ569502, AB379667) used as the outgroup are not shown; bootstrap values from 1000 replicates shown only above 60%

Furthermore, a ~900–1000 bp *cox*1 fragment was obtained from all piroplasm-positive samples. BLAST determination of these sequences confirmed the results of *18S* fragment analysis (Table 3). Phylogenetic analysis of 768 bp of all available piroplasm *cox*1 sequences was performed. All five *B. canis* sequences were placed within the *B. canis* clade (Fig. 4), whereas six unique clones of “Theileria annae” *cox*1 obtained from two different animals formed a sister clade with those of *B. microti*.

**Fig. 5:**
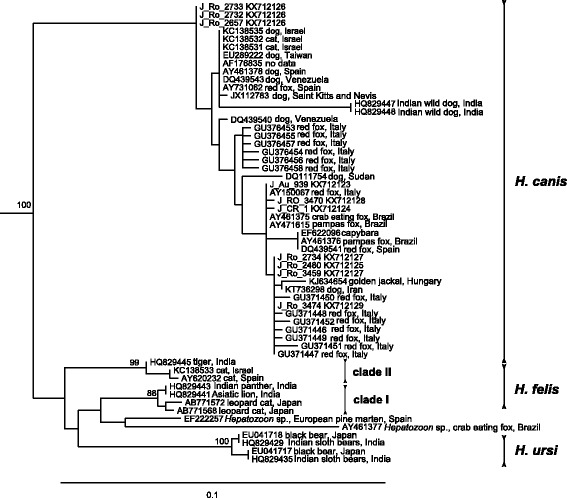
Maximum likelihood tree based on 722 nt-long alignment of 18S rDNA sequences of *Hepatozoon* spp; sequences of *Hemolivia* spp. (KF992713, KF992698, KF992702) used as the outgroup are not shown; bootstrap values from 1000 replicates shown only above 60%

A fragment of ~1700 bp of *Hepatozoon 18S rDNA* was obtained from eight Romanian jackals and from both individuals originated from the Czech Republic and Austria. Sequence analysis by BLAST confirmed 99–100% identity of *H. canis* (Table 3). In phylogenetic analysis, all 10 sequences clustered within the *H. canis* clade from dogs and other wild canids (Fig. 5). Only limited number of ~1700 bp of *Hepatozoon 18S rDNA* sequences is available in GenBank; thus shorter fragment was used for phylogenetic analysis to cover the broad geographical and host range of this parasite.

The obtained sequences of *18S rDNA* (KX712122–KX712130), *cox*1 (KX712131- KX712138) and *ITS1-5.8S* (KX712139) fragments were deposited in the GenBank database. Identical sequences were deposited under the same accession number.

## Discussion

Free-ranging canids are key hosts for a broad range of parasites including those transmitted by arthropods. The distribution and abundance of the large European carnivores (e.g. wolves, lynxes, bears) is surprisingly dynamic, showing fluctuation not only in abundance within particular areas but also in geographical distribution [5]. The gradual spread of Eurasian golden jackals is remarkable from an ecological perspective insofar as this mesocarnivore species is a predator historically largely new in central European ecosystems [6–8, 35]. The role of jackals is interesting also from an epidemiological perspective, given that they can act as reservoir hosts for several pathogens transmitted by arthropod vectors [10–16].

Our study revealed a significant proportion (70%) of examined jackals testing positive for *H. canis.* Although typically found in dogs in the Mediterranean region within the range of its only known vector, *R. sanguineus* (*s.l*.) ticks the parasite is common in red foxes but rare in dogs in areas where *R. sanguineus* (*s.l*.) is absent [4, 36]. We detected *H. canis* in golden jackals both inside and outside the *R. sanguineus* (*s.l*.) range in Romania (as illustrated in Fig. 1), and in both animals from Austria and the Czech Republic. Previously, the pathogen has been reported in golden jackals from Austria and Hungary [13, 14]. All sequences of *18S rDNA* obtained from jackals in our study cluster intermixed with sequences from dogs and foxes available in GenBank. Despite the observed heterogeneity of the obtained sequences, the analysis did not reveal any host- or geography-related pattern in the sequences’ clustering suggesting ongoing transmission between involved carnivore species.

Although piroplasms have been reported in other jackal species/lineages from Africa and Asia [37, 38], surprisingly no records exist of piroplasms in Eurasian golden jackals in Europe. To overcome the low sequence variability of the *18S* gene of piroplasmids, the amplification of partial mitochondrial *cox*1 gene in all piroplasm-positive samples was performed. The *cox*1 sequences and resulting phylogenetic analyses indicated a significantly higher degree of sequence heterogeneity and confirmed the presence of *B. canis* and “T. annae” in samples examined in this study.

We detected *B. canis* only in jackals from Romania, where the origin of positive animals corresponds with the known distribution of *D. reticulatus* [39] as well as with reported clinical cases of canine babesiosis [40–42] (Fig. 1). Phylogenetic analysis separated the clade of *B. canis* into two lineages corresponding with the previously described genotypes “A” and “B”. All five sequences from golden jackals from our study cluster within the genotype B carrying the AG nucleotides at positions 609–610 (according to AY072926) [32, 33]. The amplification of ~1700 bp *18S rDNA* fragments was successful in two *B. canis*-positive animals only, probably due to co-infection and unspecific parallel amplification of *H. canis 18S rDNA* and despite our attempts to optimize PCR conditions.

Our study has suggests the Eurasian golden jackal to be a novel host for “Theileria annae”, however, the final proof of their reservoir role requires also other than DNA-based data. This piroplasmid is widespread all over Europe in red foxes and occasionally causes clinical piroplasmosis in dogs [20, 43]. The species belongs to a basal piroplasmid clade referred to either as clade VIII *sensu* Lack et al. [44] or clade I *sensu* Schnittger et al. [34], distant from “typical” *Babesia* and *Theileria*. Generic placement of the taxon described as *T. annae* [43] is unresolved and the clade rather represents a separate genus [44]. The nomenclature of this species is recently under the discussion [45, 46] and to prevent further confusion, we refer this species to as “Theileria annae” throughout our paper. The importance of Eurasian golden jackals in this piroplasmid’s transmission cycle remains questionable, it is well possible that they, like dogs, are only accidental hosts while red foxes remain its major natural host and reservoir [20, 43].

From the epidemiological perspective, jackals in the studied area host the range of tick species typical for other carnivores including domestic dogs. The same set of individuals of Romanian Eurasian golden jackals was surveyed recently, reporting *Ixodes ricinus*, *I. hexagonus*, *Dermacentor reticulatus*, *Haemaphysalis punctata*, *H. concinna* and *Rhipicephalus sanguineus* (*s.l*.) [47]. However, the vectorial competence of individual species of ixodids parasitizing jackals deserves further attention, especially related to transmission of *Hepatozoon canis* in non-*Rhipicephalus* areas.

The only jackal, which tested positive for *L. infantum,* originated from southern Romania, close to the Bulgarian border (Fig. 1). Although Romania has not been regarded in the last 80 years as endemic for canine leishmaniasis, two recent reports of this parasite in dogs brought the disease to our attention [48, 49]. The prevalence of *L. infantum* in our study is relatively low (1/36), and it remains to be clarified whether the apparent recent re-emergence of canine leishmaniasis in Romania might be related to jackal expansion and its role as a known reservoir [15, 16].

## Conclusion

As a close relative of the domestic dog, the Eurasian golden jackal apparently hosts a wide spectrum of vector-borne pathogenic protists that affect dogs in Europe. Expanding populations of jackals rapidly colonize new localities in central Europe, and the vagrant animals may play a significant role in spreading *B. canis* into new localities in which *D. reticulatus* is present. The jackals’ role in the epidemiology of “T. annae” and *H. canis* is probably similar to that of red foxes and should be taken into account in further research on these parasites. Despite the low prevalence of *L. infantum* in our study, migrating jackals could represent a significant risk for spreading canine leishmaniasis into new areas.
